# Editorial: Dysregulation of Autonomic Cardiac Control by Traumatic Stress and Anxiety

**DOI:** 10.3389/fpsyg.2016.00945

**Published:** 2016-06-21

**Authors:** J. P. Ginsberg

**Affiliations:** Dorn Research Institute, Dorn VA Medical Center, US Department of Veterans AffairsColumbia, SC, USA

**Keywords:** autonomic nervous system, traumatic stress, heart rate variability, Hrv biofeedback, cardiac psychology

“Disorders of arousal” were defined in the past in terms of brain-based hypersensitivity to environmental stimulation (Gellhorn, 1968). Normally balanced sympathetic and parasympathetic branches of the autonomic nervous system (ANS) was historically described as “autonomic tuning,” in contrast to the disorders of arousal which are characterized by ANS dysfunction, affective lability, anxiety, stress, and emotional disorder (Gellhorn and Loofburrow, 1963; Everly and Lating, 2012). Recent studies of the relevance of the ANS to stress and mental disorders are becoming increasing in number^1^ (Agorastos et al., 2015). ANS dysregulation impacts on both physical (increasing cardiovascular risk) and mental (compromising psychological well-being) health at multiple levels. Loss of regulation of normal autonomic control of cardiac adjustment to environmental stressors thus leads to negative impacts on physiological function affecting arterial blood pressure, heart rate and rhythm, and vagal afference. Allostatic load is a term that has been used for decades to describe “the wear and tear on the body” which grows over time when the individual is exposed to repeated or chronic stress (McEwen and Stellar, 1993). Allostatic load is the physiological consequence of chronic exposure to fluctuating or heightened neural or neuroendocrine response that results from repeated or chronic stress. Thus it is, chronic autonomic imbalance finally leads to allostasis of affective, cognitive, and behavioral level of function. While the older “Disorders of Arousal” model continues to be prevalent in the scientific and clinical literature on stress-related pathogenesis, the field of stress disorders has taken great strides forward in terms of the NIMH-supported Research Domain Criteria (RDoC) initiative^2^. RDoC has successfully redefined “mental disorders” as “brain circuit disorders” using a matrix to organize increasing units of levels of analysis (genes through self-report and paradigms) for studying precisely defined domain constructs describing behavior. Most importantly, is that application of the RDoC approach brings into focus new therapeutics and new targets of therapeutics for brain disorders (Insel and Cuthbert, 2015).

This is exactly where the articles that comprise the *Frontiers Research Topic: “Dysregulation of autonomic cardiac control by traumatic stress and anxiety”* fit into clinical practice and research. Research data over the past two decades are showing that heart rate variability (HRV) is diminished in anxiety and stress-related disorders including PTSD, and lower HRV signals a disturbance of normal autonomic function and allostatic sympathetic hyper-arousal. Furthermore, there is burgeoning and very recent research showing that HRV Biofeedback—the training of resonant frequency breathing, attentional focusing, and positive emotional state—reduces symptoms of anxiety, stress, and PTSD through normalization of autonomic function. HRV biofeedback (HRVB) enables self-regulation of peripheral autonomic state using feedback to the central nervous system circuits that control emotional, cognitive, and sensorimotor activity. The study of HRV and effects of HRVB provide important insights into the mechanisms of autonomic arousal in normal, successful adaptation, and pathological states such as PTSD and anxiety.

All of the individual articles in this ebook can thus be placed into one of three groupings. The papers by Fred Shaffer et al. and Rollin McCraty and Maria Zayas share a descriptive aspect of normal autonomic regulation of cardiac function in the healthy adult human being. Kuan-Hua Chen's contribution is experimental data on fractionation of stages of parasympathetic responding during habituation to repetitive acoustic startle touches on the fundamental process of orienting vs. defensive responses in children, and provides a bridge between early and current perspectives on how derangement of autonomic “tuning” underlies disorders of arousal. Slow habituation of startle to aversive stimuli is a likely factor in development of traumatic stress, which makes for a segue into Susan Wood's paper on biomarkers of autonomic dysregulation in a rodent stress model (the only translational paper in the collection); Wood's review builds the central framework of dysregulation of autonomic cardiac function in anxiety and stress.

All of the papers in the second grouping are connected by their focus on the role of autonomic cardiac *dysregulation* underlying the development and expression of stress in humans. Results of the Steffen et al. clinical research indicate that, with careful monitoring, students receiving psychotherapy at a college counseling center had higher and sustained cortisol and heart rate (HR) during recovery after a clinically-controlled stressor. Importantly, these researchers delved deeper to find that psychotherapy participants experiencing clinically significant levels of distress during the stressor displayed elevated blood pressure and HR, concluding that physiological reactivity to stress is important for psychotherapy clients. Lee et al. calling for the RdoC approach, argue that medication and illicit substance dependence in patients with high-arousal disorders which they associate with a “parasympathetic freeze state” represent output effects of frontal asymmetry activation on *feeling* and a consequent drive for arousal attenuation. Then, Gillie and Thayer use carefully and thoughtfully selected data to bring the argument back to the well-accepted model of executive function and autonomic interactions, based on Thayer's Neurovisceral Integration model of Central Autonomic Networks (CAN) and Anterior Executive Region (AER), and effectively tie it all to PTSD. The groundbreaking thesis, that aging as indexed by poor long-term physical health outcomes and substance abuse, is presented by Williamson et al. Lastly, Conder and Conder extend the utility of HRV as a therapeutic target to treatment of concussion, a topic that is rapidly expanding in importance.

The third group contains overview considerations of the utility of understanding autonomic factors in individual health. Psychobiologic sensors are “a perfect storm” of convenience, affordability, and efficiency, Rob Drury writes; they continue to develop in sophistication of analysis of naturalistic environments and the autonomic responses of individuals, providing new and exciting avenues for health monitoring. Two of the most widely known and respected authorities in the field of HRV Biofeedback, Paul Lehrer and Richard Gevirtz, lay down a blueprint of the inner workings of this integrative medicine intervention. Finally, my colleagues and I (Ginsberg et al.) offer a bird's eye view of the history and current state of the field of study of autonomic cardiac regulation of anxiety and stress, under the unifying term Cardiac Psychology.

This Research Topic is presented with the hope that readers will find it enlightening, stimulating, and a contribution to advancement of the field.

## Author contributions

The author confirms being the sole contributor of this work and approved it for publication.

### Conflict of interest statement

The author declares that the research was conducted in the absence of any commercial or financial relationships that could be construed as a potential conflict of interest.

## Footnotes

^1^Nagai, Y. (2014). Research Topic: The relevance of the autonomic nervous system to neurological and psychiatric disorders. *Front. Behav. Neurosci*. Available online at: http://www.frontiersin.org/autonomic_neuroscience/researchtopics/The_relevance_of_the_autonomic/2333

^2^National Institute of Mental Health. NIMH Research Domain Criteria (RDoC). Available online at: http://www.nimh.nih.gov/research-priorities/rdoc/nimh-research-domain-criteria-rdoc.shtml
